# Auditory evoked neuromagnetic response latency is associated with language ability in preschoolers with an elevated likelihood of intellectual or developmental disability

**DOI:** 10.3389/fnint.2025.1585567

**Published:** 2025-05-23

**Authors:** Yuhan Chen, Lauren Young, Mina Kim, Shannon Watson, Victoria Kaufman, Bethany Beal, Ilona Tuomi, Bekah Wang, Donna M. McDonald-McGinn, J. Christopher Edgar, Emily S. Kuschner, Timothy P. L. Roberts

**Affiliations:** ^1^Lurie Family Foundations MEG Imaging Center, Department of Radiology, Children’s Hospital of Philadelphia, Philadelphia, PA, United States; ^2^Department of Radiology, Perelman School of Medicine, University of Pennsylvania, Philadelphia, PA, United States; ^3^22q and You Center, Clinical Genetics, Division of Human Genetics, Children's Hospital of Philadelphia, Philadelphia, PA, United States; ^4^Department of Pediatrics, Perelman School of Medicine, University of Pennsylvania, Philadelphia, PA, United States; ^5^Department of Psychiatry, Perelman School of Medicine, University of Pennsylvania, Philadelphia, PA, United States

**Keywords:** MEG, M50, IDD, language, preschool children

## Abstract

**Introduction:**

We have shown that a delayed auditory cortex neural response is associated with language ability in school-age children with autism spectrum disorder and related syndromes, with this delay exacerbated in the context of co-occurring intellectual disability (ID). As a clinical diagnosis of ID is generally not made until school age, identification of neural measures that precede a behaviorally assessed ID diagnosis would help identify young children likely to benefit from early treatment. The present study evaluated if the speed of auditory cortex neural activity (M50 latency) would predict language ability in 3-year-old children who have an existing diagnosis that is a risk factor associated with a range of later functional outcomes, including ID or developmental delay (DD), irrespective of autism spectrum disorder diagnosis.

**Methods:**

Thirty 3-year-old children with elevated likelihood for ID or DD (ID/DD-EL) were enrolled. Evaluable magnetoencephalography (MEG) data as well as language and cognitive ability measures were obtained from 23 participants.

**Results:**

A longer time to encode auditory stimuli (i.e., a delayed M50 cortical evoked response) in the left hemisphere predicted lower language ability. Left M50 latency was not associated with cognitive ability. Right hemisphere M50 latency was not associated with language or cognitive ability.

**Discussion:**

Present observations demonstrate that non-invasive brain imaging in conjunction with a passive auditory task (with early primary/secondary auditory cortex neural responses) can identify paths for variable language outcome in preschool children with ID/DD-EL. This lays the foundation for further investigation of these neural mechanisms as early indications for treatment as well as early signals of response to treatment.

## Introduction

Auditory evoked latencies are delayed in school-age children with autism spectrum disorder (autism, hereafter) (Edgar et al., 2015a; Roberts et al., 2010), with such delays exacerbated in the context of co-occurring intellectual disability (ID). In particular, studies from our laboratory have identified in minimally verbal/non-verbal (MVNV) school-age children with autism associations between auditory cortex neural activity and both general cognitive ability and domain-specific language/communication ability (Roberts et al., 2019). A clinical diagnosis of ID is typically not made until school age, and a limitation of research in this area is identifying preschool children with an increased likelihood of a later ID diagnosis. This is of interest, as identifying early neural mechanisms that are differentially associated with variable language and cognitive outcomes in children with elevated likelihood of ID or developmental delay (ID/DD-EL) would help detect young children likely to benefit from early targeted treatment, as well as identify early signals of response to such treatment.

Multiple genetic conditions confer a risk of intellectual disability, specifically language impairment, with functional outcomes ranging from profoundly impaired to within the average range. Genetic risk factors for intellectual disability have been identified (e.g., 22q11.2 deletion syndrome and Down syndrome), with large variability in language and cognitive ability among children with known genetic diagnoses. For example, in a large group of individuals with 16p11.2 deletion, the group *average* Full-Scale IQ (FSIQ) was in the low average range, with only 10% meeting the criteria for an ID diagnosis (Hanson et al., 2015). In Fragile X syndrome, IQ spans the full distribution of cognitive ability and varies with the extent of FMR1 protein expression (Tassone et al., 1999). Cognitive variability among individuals with Prader–Willi syndrome is less etiologically clear and thus less predictable (Whittington and Holland, 2017).

Language impairment and an ID/DD diagnosis are also common in children with birth and prenatal complications. Similar to children with a genetic syndrome, there is substantial variability in language outcome in children born preterm (<34 weeks gestational age), with some children showing no language delay, some presenting with an early language delay but catching up to age-appropriate levels by school age, and some presenting with persistent language difficulties (Belardi et al., 2017; Chilosi et al., 2019; Dale et al., 2003; Rescorla, 2002).

The field has little insight into the neural mechanisms associated with the severity of impairment in preschool children with an elevated risk for ID/DD. Identifying valid and replicable biomarkers across heterogeneous populations (Jeste et al., 2015; Kozak and Cuthbert, 2016; Miller et al., 2016; Yee et al., 2015) that are associated with language ability before the start of kindergarten would identify children at-risk for ID/DD likely to benefit from early intervention, as well as to monitor and potentially predict treatment response. Identifying neural biomarkers *across* a broad range of ID/DD-EL etiologies rather than *within* a specific ID/DD-EL diagnostic group is needed, as traditional diagnostic categories are sometimes too limited or heterogeneously large for biological phenomena (Miller et al., 2016). Given that different supports are needed for young children with different severities of language and cognitive impairments or children who have language delay without cognitive impairment, identifying neural markers that are differentially sensitive to general cognitive ability versus language-specific ability would inform the development of targeted intervention plans.

Findings from our laboratory and others have shown atypical auditory M50 and M100 responses, recorded using magnetoencephalography (MEG), in school-age children with autism without cognitive impairment, as well as children with autism who are MVNV. These findings include delayed superior temporal gyrus (STG) auditory responses (Edgar et al., 2015a; Edgar et al., 2014; Gage et al., 2003; Naatanen and Picton, 1987; Roberts et al., 2012; Roberts et al., 2010; Roberts et al., 2019), reduced STG 40-Hz auditory steady-state total power (Wilson et al., 2007), pre-and post-stimulus pure tone STG low-and high-frequency oscillatory abnormalities (Edgar et al., 2015a), and atypical hemispheric lateralization of auditory responses (Stroganova et al., 2013).

Using procedures developed in our laboratory (Kuschner et al., 2021), M50 auditory measures have been examined in school-aged children with autism who are MVNV as well as in a broad range of children with IDD, such as Down syndrome, Bainbridge–Roper syndrome, NAA10 gene mutation, and 8p23.3 deletion (Kuschner et al., 2021; Roberts et al., 2024; Roberts et al., 2019). Roberts et al. (2019) showed that MVNV children with autism had a later M50/M100 latency compared to age-matched children with autism whose IQs were in the average range, even though in both cohorts, a later auditory evoked response latency was associated with worse language ability. Of note, MVNV children with autism often have significant cognitive impairment, with the neurophysiological mechanisms underlying language ability, independent of cognitive processes, not yet understood.

To better understand the development of auditory processes in children before they receive a diagnosis of ID/DD, the present study sought to ascertain the discriminatory potential of auditory evoked neuromagnetic response latencies with respect to language and cognitive ability in a cohort of 3-to 4-year-old children with an existing diagnosis that puts them at an increased likelihood for ID/DD. A passive auditory task was used, as such tasks provide the ability to assess auditory cortex neural function across the lifespan and a range of behavioral competence (Chen et al., 2023; Edgar et al., 2015b; Roberts et al., 2019). As the earlier auditory M50 response is more prominent than the later M100 response in younger children (Edgar et al., 2020), the present study focused on source-localized left and right hemisphere M50 auditory responses.

## Materials and methods

### Participants

Thirty children with ID/DD-EL were enrolled and participated in MEG and language and cognitive assessment sessions. Inclusion criteria were: (1) male or female, aged 2 years 8 months to 4 years 5 months; (2) an existing early childhood diagnosis associated with an increased likelihood of later diagnosis of ID (e.g., known genetic differences linked to IDD such as Down syndrome or 22q11.2 deletion syndrome, autism spectrum disorder, global developmental delay, neurological conditions such as cerebral palsy and prematurity) or documented developmental delay (IQ < 85); and (3) English as the primary language spoken in the home. Exclusion criteria included: (1) diagnosis of uncorrectable vision impairment (clinical blindness); (2) permanent hearing loss of greater than 25 dB; and (3) use of an implanted hearing aid. The study was approved by the Children’s Hospital of Philadelphia IRB, and all families provided written consent.

### Language and cognitive assessment

For each child, language ability was assessed using the Preschool Language Scale, Fifth Edition (PLS-5) (Zimmerman et al., 2011). PLS-5 total score raw scores were obtained from the sum of receptive and expressive subscale raw scores. Cognitive ability was measured either via the full-scale IQ score from the Wechsler Preschool and Primary Scale of Intelligence Fourth Edition (WPPSI-IV) (Wechsler, 2012) or the cognitive domain standard scores from the Bayley Scales of Infant and Toddler Development Fourth Edition (Bayley-4) (Bayley and Aylward, 2019). Children who were unable to establish a baseline on the WPPSI-IV were administered the Bayley-4 Cognitive domain to ensure a developmentally appropriate assessment.

### MEG data acquisition and auditory examination

Whole-head MEG data were obtained in a magnetically shielded room (Vacuumschmelze GmbH & Co. KG, Hanau, Germany) using a 275-axial gradiometer system (CTF, Coquitlam, BC) and with synthetic third-order gradiometer noise correction applied. MEG data were acquired at a sampling rate of 1,200 Hz. Before MEG acquisition, three head position indicator (HPI) coils were placed to serve as landmarks on the child’s head. The child’s head shape, anatomical landmarks (nasion, right and left preauricular points), and locations of the three HPI coils were digitized using a FastSCAN System (Polhemus, Colchester, VT, USA). The child’s head position was monitored using the three HPIs attached to the scalp with continuous head localization (essentially the magnetic detection of alternating current electrical activation of the HPI coils at non-harmonic frequencies distant from the range of brain activity). A research assistant with experience scanning young children accompanied the child and helped the parent keep the child calm and alert during the examination. A variety of strategies were used to engage the child during the scan (see details in Chen et al., 2021), including projecting an age-appropriate silent video on a screen placed above the child’s head (viewing distance 55 cm) as well as providing toys to maintain the child’s attention and focus. For each participant, otoacoustic emission (OAE) testing was performed on the same day as the MEG examination.

Auditory stimuli consisted of 500 Hz sinusoidal tones 300 ms in duration, with 10 ms onset/offset ramps. The offset-to-onset interstimulus interval varied between 600 and 2,000 ms. Stimuli were presented via a free-field speaker (Panphonics Sound Shower, Turku, Finland) at 85 dB SPL, and the distance between the free-field speaker and each participant was the same. MEG recordings were obtained while the child was awake and alert. The auditory examination lasted 8 min (~300 trials).

### Magnetic source analyses

MEG data were analyzed using Brainstorm^1^ (Tadel et al., 2011). For each child, digitized surface points from FastSCAN representing the shape of the child’s head (>10,000 points) were used to co-register each child’s MEG data to an age-appropriate MRI template (O’Reilly et al., 2021; Richards et al., 2016) using an affine transformation that accommodated global scale differences between the child’s anatomy and the atlas.

All MEG data were band-pass filtered from 3 to 55 Hz (transition bands 1.5–3.0 Hz and 55–63.25 Hz) and with a 60 Hz power-line notch filter applied. Heartbeat and eyeblink artifacts were removed via independent component analyses (ICA). Trials with MEG activity exceeding 500 fT, due to excessive motion or magnetic noise artifact, were eliminated. Other artifacts (e.g., movement and muscle artifacts) were visually identified and removed. Artifact-free auditory evoked responses were obtained by averaging evaluable epochs from 200 ms pre-to 500 ms post-stimulus. Across all children, an average of 274 artifact-free trials were obtained. Using the auditory evoked response, whole-brain activity maps were computed using minimum norm estimation (MNE; Hämäläinen and Ilmoniemi, 1994; Hauk, 2004; Lin et al., 2006; Matsuura and Okabe, 1995), with minimum-norm imaging estimating the amplitude of brain sources constrained to the cerebral cortex and current dipoles normally oriented to the local cortical surface (Baillet et al., 2001). The auditory evoked-response activity was mapped to each child’s cortical-surface source space (~15,000 vertices; varied across infants) as a function of time. For each child, an MEG noise covariance matrix was obtained from an empty room recording obtained immediately after the child’s scan. MNE solutions were computed with normalization as part of the inverse routine, based on the noise covariance.

**Figure 1:**
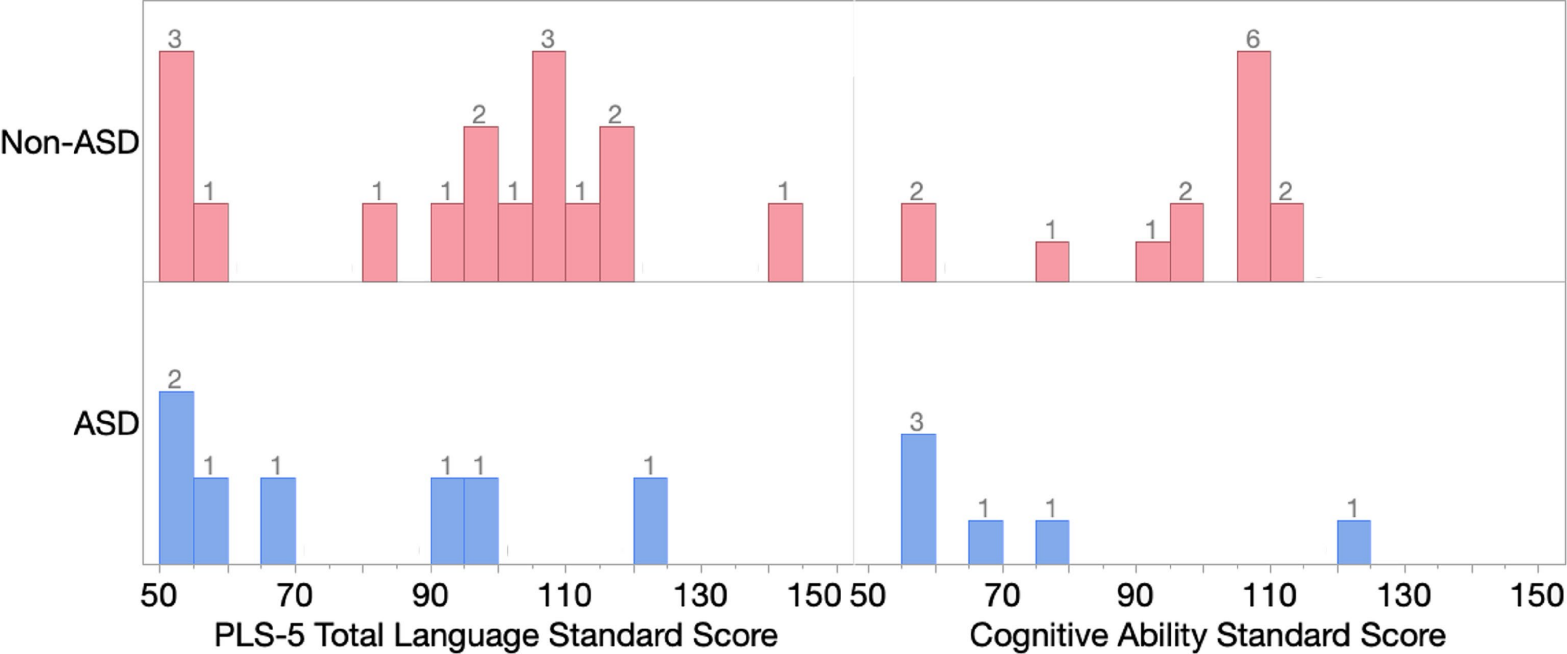
Histogram of PLS-5 total language standard scores and cognitive ability standard scores in ID/DD-EL children with and without an autism diagnosis. The numbers shown in the histogram indicate the number of participants for each score range.

In each child, left-and right-hemisphere auditory source timecourses were obtained from left-and right-hemisphere auditory ROIs (Chen et al., 2023). The M50 response was first identified via the sensor magnetic field sink and the source topography (e.g., in the left hemisphere, an anterior source and posterior sink) and with left and right M50 ROIs created at the time of the peak M50 sensor activity using an amplitude threshold of 5 nAm and a cluster threshold of ≥50 neighboring cortical surface vertices. A left and right auditory source timecourse was obtained via averaging across vertices within the left and right ROI, with post-stimulus activity normalized to the baseline (i.e., providing post-stimulus Z-score measures; see Figure 1, auditory source timecourses from two representative participants). Using in-house MATLAB software, left and right peak M50 latencies, within ±30 ms surrounding the peak time point identified at the sensors, were identified in source space.

### Statistical analysis

Statistical analyses were conducted using JMP statistical software (SAS Institute Inc., 2021). The present study hypothesized that the auditory M50 latency may serve as a proxy for language and/or cognitive ability. Sex was also considered a confounder/mediator. Structural equation models (SEMs) were used to evaluate whether left and right auditory M50 response latencies predicted language and cognitive scores, respectively, while recognizing the possible influence of sex. Our models include the following paths: M50 latency predicts language/cognitive scores, sex predicts language/cognitive scores, and sex predicts M50 latency. Four SEMs were specified: Models 1 and 2 estimated the correlation between M50 latency (separate for left and right hemispheres), sex, and PLS-5 score (as an index of language ability). Models 3 and 4 estimated the correlation between M50 latency (separate for left and right hemispheres), sex, and cognitive standard score (as an index of general cognitive ability). Akaike Information Criteria (AIC) values were computed to estimate the goodness of fit of each model, with lower AIC values indicating a better-fit model.

Given that the literature has shown evidence of sex differences in language development during early childhood (see review in Rinaldi et al., 2023), we further explore whether M50 latency accounts for significant variance in language scores between female and male participants. A linear mixed model was run with the PLS score entered as a dependent variable and with M50 latency, age, sex, and the M50 latency by sex interaction term entered as fixed effect variables.

## Results

Of 30 enrolled children, evaluable MEG data were available from 23 children. MEG datasets from seven children were not available/excluded due to the child being unable to tolerate the scan (*N* = 4), magnetic noise artifact (*N* = 1), or evaluable data that lacked auditory evoked response (*N* = 2). All children with evaluable MEG data have completed the PLS-5 assessment. Of 23 children, 13 have completed WPPSI-IV, 7 completed the Bayley-4 cognitive subscale, and 3 could not complete WPPSI-IV or Bayley-4. Tables 1, 2 shows the descriptive statistics of age, language scores, cognitive scores, and left and right auditory M50 latency between (A) female and male participants and (B) participants with and without a diagnosis of autism. Welch’s t-tests indicated that no difference was observed between male and female participants or between participants with and without autism, in any of the Table 1 measures.

**Table 1:** Descriptive statistics between females and males.

Mean (SE) Range (Min–Max)	Female (*N* = 9)	Male (*N* = 14)	*t*-test	All (*N* = 23)
Age (months)	39.98 (1.28) 37.73–42.17	41.63 (4.72) 33.03–53.53	*t* (16) = 1.24 *p* = 0.23	40.99 (3.80) 33.03–53.53
PLS-5 Total raw score	70.78 (8.10) 18–90	60.57 (7.09) 25–105	*t* (18) = −0.95 *p* = 0.36	64.57 (5.34) 18–105
PLS-5 Total language standard score	95.11 (25.06) 50–118	82.50 (29.29) 50–141	*t* (18) = −1.10 *p* = 0.28	87.43 (27.83) 50–141
Cognitive ability standard score	94.12 (8.67) 55–114	85.25 (7.06) 55–121	*t* (15) = −0.79 *p* = 0.44	88.80 (5.42) 55–121
Left M50 Latency (ms)	138 (4.12) 122–161	138 (10.90) 115–189	*t* (21) = 0.14 *p* = 0.89	138 (3.33) 115–189
Right M50 Latency (ms)	139 (4.91) 103–217	143 (4.11) 124–184	*t* (10) = 0.38 *p* = 0.71	142 (4.81) 103–217
Accepted number of trials	305 (33) 152–514	254 (22) 89–342	*t* (15) = −1.30 *p* = 0.21	274 (19) 89–514

**Table 2:** Descriptive statistics between children with and without ASD.

Mean (SE)Range (Min-Max)	ASD (*N* = 7)	Non-ASD (*N* = 16)	*t*-test
PLS-5 Total language standard score	75.86 (27.16) 50–120	92.50 (27.41) 50–141	*t* (11.61) = 1.35 *p* = 0.20
Cognitive ability standard score	71.67 (25.94) 55–121	96.14 (20.16) 55–114	*t* (7.72) = 2.06 *p* = 0.07
Left M50 Latency (ms)	143 (25.41) 115–189	135 (9.96) 123–154	*t* (6.82) = −0.73 *p* = 0.49
Right M50 Latency (ms)	145 (8.69) 127–154	140 (27.29) 103–217	*t* (20.07) = −0.61 *p* = 0.55

Figure 1 shows the distribution of language and cognitive standard scores within our cohort, separately for children with and without an autism diagnosis. Of note, the majority of children in our cohort fall within the normal range of language and cognitive ability (e.g., 75% of ID/DD-EL children without autism and 43% of ID/DD-EL children with autism have PLS-5 standard scores >85; see Figure 1).

### Left and right hemisphere auditory M50 latency

**Figure 2:**
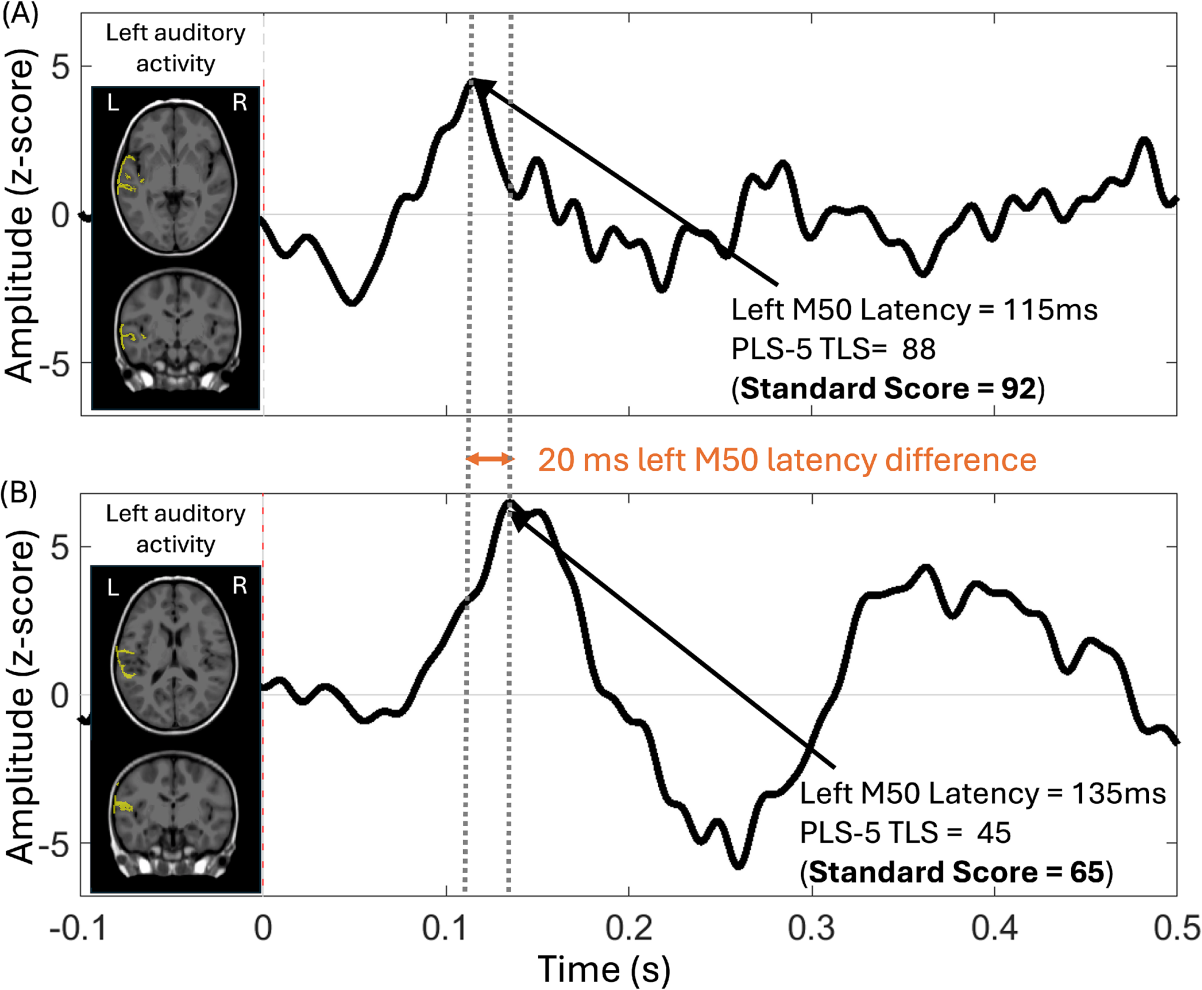
Auditory source timecourse from the left auditory cortex in two ID/DD-EL children, along with the anatomic source overlaid (yellow) on axial and coronal MRI views. The auditory source timecourse was obtained via averaging across vertices within the left auditory cortex ROI, with post-stimulus activity normalized to the baseline (auditory response amplitude expressed in Z-score on the y-axis). **(A)** The auditory cortex response from a child (52.8 months old) with language performance in the average range (PLS-5 Total Language Score (TLS) = 88, Standard Score = 92) is characterized by a relatively earlier M50 response. **(B)** The auditory cortex response from a child (42 months old) with language performance 2 SDs below average (PLS-5 TLS = 45, Standard Score = 65) is characterized by a relatively later M50 response.

The auditory M50 response component was the earliest positive response, peaking ~100–150 ms post-stimulus in young children. In children with ID/DD-EL, there was large variability in left and right hemisphere auditory M50 latency (see Tables 1, 2), consistent with broad variation in language and general cognitive abilities. Figure 2 shows auditory source timecourses from two representative children with ID/DD-EL—a child with relatively early left M50 latency and with average language performance (standard score = 92) on the PLS-5 (top panel) and a child with relatively late left M50 latency and a language score 2 SD below average (standard score = 65) on the PLS (bottom panel).

### Predictors of language and cognitive ability

**Figure 3:**
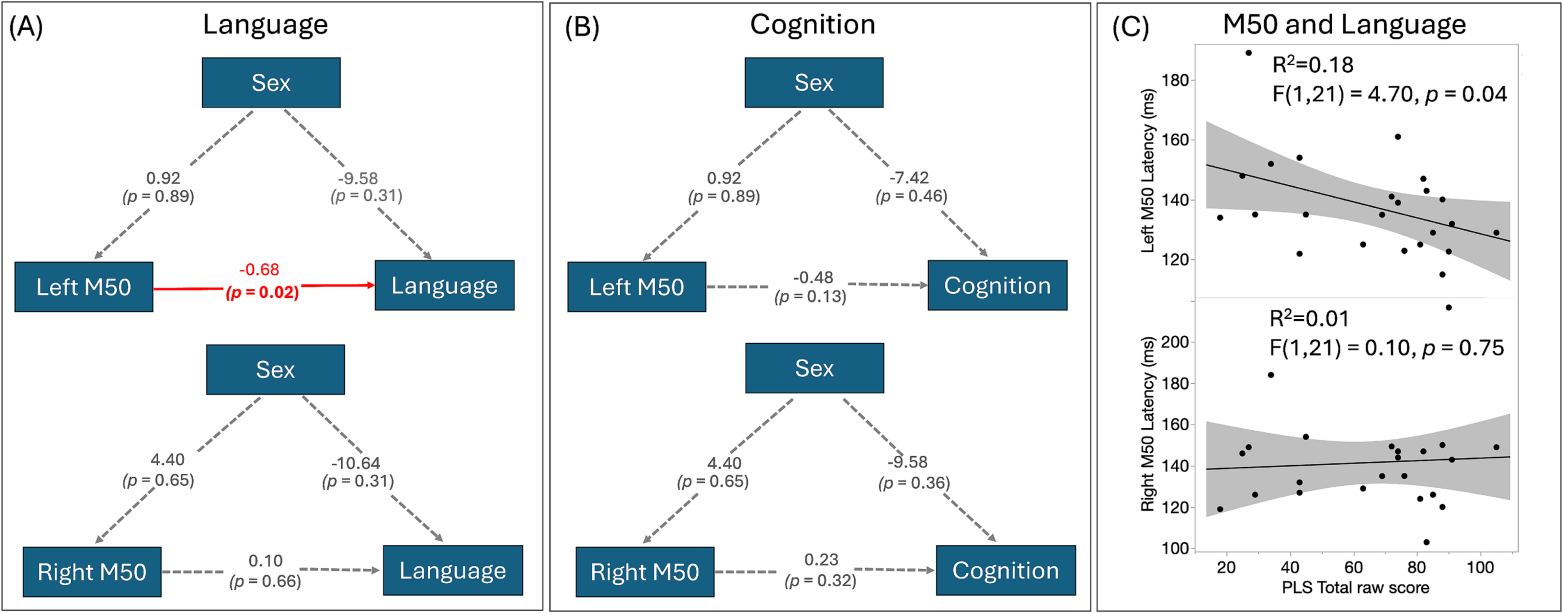
**(A)** SEMs of M50 latency, sex, and language. The estimates of the regressions between the factors are shown. Directed paths are shown as arrows between the factors, with a significant association highlighted in red; **(B)** SEMs of M50 latency, sex, and cognition show no significant associations; **(C)** The scatterplot between left and right M50 latency and PLS-5 score identifies a significant association between left hemisphere M50 latency and language ability, with a later M50 latency associated with a lower language ability score.

Figure 3 shows the correlations between left and right M50 latency and PLS-5 and cognitive scores (Figures 3A,B). Only left M50 latency predicted language scores. The AICs for all four models are within a similar range as the following: 463 (left M50, sex, language), 484 (right M50, sex, language), 436 (left M50, sex, cognition), and 454 (right M50, sex, cognition). Neither left nor right M50 latency predicted cognitive scores. Sex did not mediate the relationship between M50 latency and PLS-5 or cognitive score. Figure 3C shows associations between left (top) and right (bottom) M50 latency and PLS-5 score. Of note, the standard least square test indicated language scores were significantly associated with cognitive scores (*R*^2^ = 0.86, *F*(1, 18) = 109.66, *p* < 0.0001).

To examine differences in M50 latency and language scores associations between females and males, linear mixed model results showed an M50 latency by sex interaction (*F*(1,40) = 5.33, *p* = 0.026), with simple-effect analyses showing a relationship between M50 latency and language scores only in males.

## Discussion

The present study evaluated the ability of auditory cortex neural activity to predict language and cognitive performance in 3-year-old children with ID/DD-EL. Left-hemisphere M50 latency was associated with language scores, with left M50 latency being sensitive to language but not cognitive scores. The non-significant trend between left M50 and cognitive ability can largely be accounted for by the fact that cognitive and language measures were highly correlated. The following text discusses M50 latency findings with respect to previous studies in this area.

### Left hemisphere auditory M50 latency predicts language ability in children with ID/DD-EL

M50 latency indicates the time it takes auditory information to travel from the ear to the primary/secondary auditory cortex. M50 is the earliest “middle latency” cortical response identified by electrophysiological methods and is the magnetic counterpart of the mature ERP P1 component (for an extended discussion, see Chen et al., 2023; Edgar et al., 2020). Consistent with the previous literature (Kuschner et al., 2021; Roberts et al., 2024; Roberts et al., 2019), our findings show left but not right hemisphere M50 latency and language associations, with a later (more delayed) left M50 latency associated with lower language scores. Present results are consistent with studies exploring the relationship between auditory cortex neural activity and language in children 2–6 years old (Yoshimura et al., 2012; Yoshimura et al., 2014). Additionally, some studies have shown that auditory responses in infancy predict language scores later in development (Choudhury and Benasich, 2011; Musacchia et al., 2013).

A left hemisphere specialization in language processing was observed at a young age. Research has shown that rapid sound discrimination is needed for mapping meaning onto sounds, with this ability hypothesized to be associated with the maturation of the left hemisphere auditory cortex (Hickok and Poeppel, 2007). This is perhaps mirrored in the different maturation rates of left and right auditory cortices. Findings from our and other laboratories have shown that the left auditory cortex matures more slowly than the right auditory cortex between infancy and toddlerhood (Chen et al., 2023) and across the life span (Edgar et al., 2020; Paetau et al., 1995; Parviainen et al., 2019), with a slower maturation rate of the left auditory cortex likely accounting for the associations between left M50 latency and language scores, as language learning is a slow, non-linear process that starts early in life and continues throughout adolescence and adulthood. In line with findings from older children’s studies, previous findings from our typically developing infant MEG study (Chen et al., 2023) showed that a later infant auditory cortex latency (the infant “P2m” is equivalent to the “M50” in young children and adults) was associated with lower language scores in children 12–24 months old, an age when expressive language begins to emerge. This is likely because more rapid auditory processing allows infants to more efficiently process linguistically relevant acoustic information (Benasich et al., 2002). Finally, for our cohort of preschool children with ID/DD-EL, the mixed model analysis results showed stronger associations between M50 latency and language scores in male and female participants, suggesting underlying neurophysiological differences between boys and girls that likely contribute to sex differences in early stages of language development.

Present findings extend our previous findings, showing early (3–4 years of age) hemispheric specialization of the auditory cortex in language development, even in young children with an elevated likelihood of ID. Across a wide spectrum of developmental disorders, early identification and remediation of language difficulties likely lie in understanding early auditory encoding mechanisms (Musacchia et al., 2015). As the neural mechanism of early language development is better understood in at-risk young children, the field can begin to understand why some children have only delayed language, while others have delayed language *and* delayed cognitive ability.

### Biomarkers to predict language impairment

Our laboratory has a long history of studying auditory function and language development in a wide spectrum of school-age children with autism, including verbal children without cognitive impairment, as well as minimally verbal/non-verbal children (Kuschner et al., 2021; Roberts et al., 2019). Abnormal auditory neuromagnetic M50 (and later M100) component responses reflecting primary/secondary cortex processing have been reported. Our previous findings (Roberts et al., 2019) from school-age children with autism showed a delayed M50 in both hemispheres. Present left hemisphere M50 and language associations findings indicate that in young children, abnormal auditory cortex processing is more pronounced in the left hemisphere, with this delay likely observed in both hemispheres later in development.

Present findings also indicated that a later M50 latency is sensitive to language ability, observed in both children with autism as well as in children at more general risk for cognitive impairment. Given that performance on the language and cognitive ability assessment is highly correlated, it is challenging to identify biomarkers that are specifically sensitive to language delay and independent of cognitive ability. Multi-measure batteries of tests and combinatorial measures/statistics will likely be needed.

### MEG-PLAN for preschoolers with ID/DD-EL

Neuroimaging research with young children with neurodevelopmental disorders has been limited due to barriers like tolerating the loud noises of scanning and the inability to remain still during MRI scans. MEG is a non-invasive neuroimaging technique that provides whole-head measures of neural activity with millisecond temporal resolutions, which is silent, has minimal preparation time, and has head movement compensation methods that can correct for moderate head motion, making it particularly suitable for studying brain activity in young children and children with developmental differences (Chen et al., 2019). To better understand brain function across individuals with various cognitive abilities across all ages, our team has developed MEG-PLAN (Kuschner et al., 2021), a clinical and technical protocol to obtain MEG data in minimally verbal or non-verbal neurodevelopmental pediatric populations. The present study applied MEG-PLAN in this preschool-aged cohort and yielded a 77% evaluable data scan completion rate, comparable to or slightly higher than the rate of 71% in our previous school-age cohort of MVNV children with autism (Kuschner et al., 2021). Given that the present cohort was chronologically younger and many had developmental delays, the present results are encouraging.

### Future directions and limitations

Present findings show an association between neural signatures of left hemisphere auditory processing and language. A future direction is to investigate whether auditory neural measures at a young age (e.g., 3 years old) contribute to the prediction of functional outcome in school-aged children (e.g., 6 years old), and thus at an age when a formal diagnosis of intellectual disability is usually established. Prospective follow-up of the current cohort will help answer the above.

Continued efforts to encourage and accommodate more inclusive scanning methodologies are warranted from scientific and sociological perspectives. The application of MEG-PLAN to younger children represents a promising avenue to achieve this inclusivity. The use of clinical and technical protocols such as MEG-PLAN across neuroimaging technologies will expand our understanding of neural processes across a broad range of phenotypic presentations and increase the generalization of results.

Although no sex difference was observed on M50 measures as well as language and cognitive scores, given exploratory findings of differential relationships between M50 and language ability in females and males, future studies with large sample sizes and age ranges are warranted to explore potential sex effects, particularly on the relationships between M50 latency and language scores in young children with ID/DD-EL.

In summary, non-invasive, quantitative, and hemisphere-specific assays of neural auditory processing offer insight into the physiological etiology of language impairment. They may also provide a targeted substrate for individualized interventions and serve as an objective brain marker for predicting later language outcomes.

## Data Availability

The MEG data for this project (de-identified) may be obtained via a Data Use Agreement with the Children’s Hospital of Philadelphia. Researchers interested in access to the data may contact Dr. Yuhan Chen at cheny4@chop.edu. Software for MEG data analysis associated with the current submission is available at https://mne.tools/stable/index.html and https://neuroimage.usc.edu/brainstorm/.

## Footnotes

^1^ http://neuroimage.usc.edu/brainstorm
